# Bayesian Parameter Estimation and Segmentation in the Multi-Atlas Random Orbit Model

**DOI:** 10.1371/journal.pone.0065591

**Published:** 2013-06-18

**Authors:** Xiaoying Tang, Kenichi Oishi, Andreia V. Faria, Argye E. Hillis, Marilyn S. Albert, Susumu Mori, Michael I. Miller

**Affiliations:** 1 Center for Imaging Science, The Johns Hopkins University, Baltimore, Maryland, United States of America; 2 Department of Radiology, The Johns Hopkins University, Baltimore, Maryland, United States of America; 3 Department of Neurology, The Johns Hopkins University School of Medicine, Baltimore, Maryland, United States of America; 4 Department of Physical Medicine and Rehabilitation, The Johns Hopkins University School of Medicine, Baltimore, Maryland, United States of America; 5 Department of Cognitive Science, The Johns Hopkins University School of Medicine, Baltimore, Maryland, United States of America; 6 The Johns Hopkins Alzheimer's Disease Research Center, Baltimore, Maryland, United States of America; 7 Institute for Computational Medicine, Johns Hopkins University, Baltimore, Maryland, United States of America; 8 Department of Biomedical Engineering, Johns Hopkins University, Baltimore, Maryland, United States of America; University of East Piedmont, Italy

## Abstract

This paper examines the multiple atlas random diffeomorphic orbit model in Computational Anatomy (CA) for parameter estimation and segmentation of subcortical and ventricular neuroanatomy in magnetic resonance imagery. We assume that there exist multiple magnetic resonance image (MRI) atlases, each atlas containing a collection of locally-defined charts in the brain generated via manual delineation of the structures of interest. We focus on maximum a posteriori estimation of high dimensional segmentations of MR within the class of generative models representing the observed MRI as a conditionally Gaussian random field, conditioned on the atlas charts and the diffeomorphic change of coordinates of each chart that generates it. The charts and their diffeomorphic correspondences are unknown and viewed as latent or hidden variables. We demonstrate that the expectation-maximization (EM) algorithm arises naturally, yielding the likelihood-fusion equation which the a posteriori estimator of the segmentation labels maximizes. The likelihoods being fused are modeled as conditionally Gaussian random fields with mean fields a function of each atlas chart under its diffeomorphic change of coordinates onto the target. The conditional-mean in the EM algorithm specifies the convex weights with which the chart-specific likelihoods are fused. The multiple atlases with the associated convex weights imply that the posterior distribution is a multi-modal representation of the measured MRI. Segmentation results for subcortical and ventricular structures of subjects, within populations of demented subjects, are demonstrated, including the use of multiple atlases across multiple diseased groups.

## Introduction

The advent of high-resolution T1-weighted magnetic resonance (MR) neuroimaging technologies facilitates a detailed exploration into human brain anatomy. Many quantitative studies have demonstrated that morphometric and functional responses of cortical and subcortical brain structures are highly correlated to numerous neuropsychiatric illnesses. There now exists a large research community supported by universally deployed software packages [1]–[5] applying automated methods for reconstruction of the human brain structures, which often rely on pre-defined brain atlases. These atlases represent structural and functional information of the brain associated to single-subject, population-averaged, or multiple brain atlas coordinate systems including whole brain based coordinate systems [6]–[11], white matter based coordinate systems [12]–[17], and surface based coordinate systems [18]–[21]; see [22] for an excellent review. Often these are coupled with global deformable template methods, small and large deformation in nature [23]–[38], for transferring information across anatomical coordinate systems.

In these deformable template approaches, the solutions inherit the smoothness and the topological properties from the atlas. The problem focused on in this paper is to extend the generative random diffeomorphic orbit model that has been used in single atlas approaches [30], [31], [35], [39] to the multiple atlas model, in which not only are the diffeomorphic changes in coordinates unknown but also jointly measurable parameters are unknown such as those arising in: (1) atlas labeling corresponding to disease inference, (2) structure parameters such as volumes, or (3) dense label field estimation associated with segmenting the target image into anatomically defined regions. In all the three examples, the atlas in the collection is unknown in generating the image, implying the posterior distribution is multi-modal determined by the multiple atlases. In these global deformable template methods [40], the parameters to be estimated are not “isolated” from the simultaneous acquisition of the global shape phenotype, which is encoded via the structure of the template and the associated deformation.

Since the atlases used for interpreting the image are not known, the conditional-mean technology of the expectation-maximization (EM) algorithm [41] underlies the problem. As we will show, the conditional-mean explicates the weights with which the atlases contribute to the interpretation of the image in the multi-modal representation. In this setting, there is a likelihood indexed over each atlas which is then combined via superposition to generate the single a posteriori distribution that the Bayes maximum a posteriori (MAP) estimator optimizes. The superposed weights are the conditional expectations of the latent variables determining the amount that each atlas-specific likelihood is factored into the single a posteriori likelihood. We name this the likelihood-fusion equation.

A significant extension, developed in this paper, of the random atlas model is to add to the global deformable template the notion of locality which is usually associated to the local approaches from differential geometry [42]. Here an atlas is defined as collections of *local charts* linked through diffeomorphic coordinate transformations. The anatomical model constructs the atlas via charts of subcortical and cortical volumes delineated in varying anatomical coordinate systems. In our case, we focus on subcortical structures and the ventricles. The MAP problem labels each voxel of the target image via mixtures of the locally-chart-defined conditional a posteriori probabilities. Since for any voxel, a chart from any of the atlases could be the generator of its mean field and the associated conditional a posteriori probability, the conditional-mean of the latent variables on chart selection is calculated for each voxel in the target image, thus providing locality in the segmentation as part of the global model.

The multi-atlas random orbit model used here for segmentation differs from several other approaches in the following ways. First, the proposed method solves for the single set of unknown segmentation variables 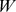
and conditions only on the observable image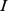. It does not generate a set of segmentation labels associated to each atlas-chart interpretation, which might then be combined via voting for fusing based on a performance metric [37], [43]–[49]. The conditional expectation framework derived here explicates the role of each chart and atlas by averaging via the conditional-expectation over the atlas-dependent log-likelihoods generating the single fused likelihood, from which the segmentation labels are generated as maximizers. This is likelihood-fusion instead of label-fusion.

Also, it is noteworthy that, in the likelihood-fusion approach, we do not generally find that the posterior probability is concentrated as a delta-function supported on one or a small number of the same atlases, which would be equivalent to the generalized likelihood problem in which the atlases and charts are tested separately with the “closest” ones determining the solution via combination as in [45], [49]–[51]. The fact that the convex combination of atlases is rarely concentrated on a single or a small subset of atlases implies that the likelihood-fusion mediates the high dimensionality of atlas selection which the generalized likelihood problem would suffer from. The likelihood-fusion is associated to averaging of log-probabilities over multiple atlases.

The method proposed here is a generative model approach, more akin to the approach suggested in [52]. The generative model we use here extends the conditionally random field orbit model of Computational Anatomy (CA) to the multiple-atlas case, modeling the images as conditionally random fields conditioned on the random segmentation field and the random unknown atlas charts to be selected. Chart selection is applied throughout the field, extending the global nature of diffeomorphic methods to local selection via spatial chart selection throughout the random field.

In this paper, we investigate the quality of the multi-atlas multi-chart diffeomorphic orbit model for the segmentation of deep gray matter structures, as well as the ventricles, using T1-weighted MR images. We were particularly interested in characterizing brain atrophy, and therefore, we tested our method in elderly and dementia populations. Results from the automated segmentation scheme have been compared with the manual segmentations to examine the accuracy of the method. More specifically, we investigate: 1) the level of accuracy we can achieve using a single-atlas approach; 2) the degree of improvement by incorporating the multi-atlas approach; and 3) the impact of the anatomical variability on accuracy based on a normal elderly and a dementia patient population.

## Methods

### 2.1 Atlas Selection and The Random Orbit Model

We first examine the class of maximum a posteriori problems in which the generalized parameters 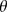 are jointly distributed with respect to the observed MRI image 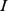 in the context of a family of atlases 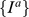. The parameters can take several forms – the disease type associated to the image, the volume of a structure in the image, or the labeling of the image into a segmentation field of subcortical structures. The likelihood model for inference based on a single atlas 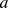 is the form of a conditional density jointly measured with the unknown parameters 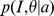. Viewing the multiple-atlas problem with atlas 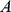 random, the fusion of the likelihood functions gives the multi-modal mixture model:
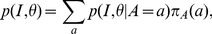
(1)with 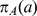 the prior averaging over atlases. This is the generative model with which we score each image and perform inference on the parameters within our multi-modal model.

### 2.1.1 The Random Orbit Model

Scoring the images in Eq. (1) boils down to the calculation of the conditional density of the image given any particular atlas 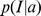. For this, we use the generative random orbit model to model the image as a random field [31], a noisy observation of an unknown change in coordinates 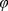 of the underlying atlases 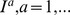 which generate it. Conditioned on the atlas as well as the diffeomorphism, the observed image has a conditional density indexed over the voxel lattice 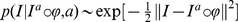, with the diffeomorphisms generated via flows 
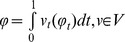
. We use the shorthand notation for the density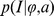.

The diffeomorphic flows are generated by the set of time-indexed vector fields 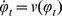 with finite integrated norm 
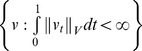
. The flows are spatially smooth since time-sections of the vector fields are of finite norm 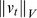 dominating a Sobelev norm of spatial derivatives existing in squared error [30]. For computational purpose, we use an operator-induced norm so that 
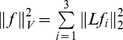
 and 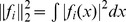
with the differential operator 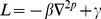, where
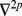
is the Laplacian operator with power 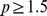, and 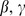
are real numbers. The prior in the random diffeomorphism orbit model is built using the geodesic energy in the space of diffeomorphisms 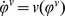
relative to the identity mapping, privileging the initial tangent vector determining the geodesic 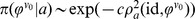 with 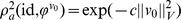. In the random orbit model, the conditional density of the image is computed via the nuisance integral:
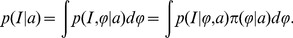
(2)


### 2.1.2 Atlas Selection for Real-Valued MAP Estimation

Model selection plays a fundamental role in the MAP estimation of parameters. We associate the high dimensional parameter set 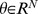 to the MRI 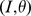. At the heart of the MAP estimation problem is how much each single atlas contributes to interpreting the image jointly with its parameters, denoted as the conditional probability according to 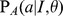. We use the EM algorithm to find the MAP estimator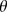.Shown in Appendix S1 is the proof that the EM algorithm is monotonic in likelihood for the sequence of segmentation labels and that fixed points satisfy the necessary conditions of being a MAP estimator.

### 2.2 The Hierarchical Segmentation Random Orbit Model

Now we examine MAP estimation in the high-dimensional setting of unknown segmentation fields, 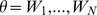 corresponding to subcortical labelings 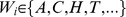
of amygdala, caudate, hippocampus, thalamus…, associated to the MRI 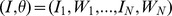 indexed over the voxel lattice of size 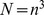.

We define a hierarchical model between the image and the underlying diffeomorphic change in coordinates of the atlas, so that 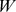 splits the target image and the diffeomorphic change of coordinates. Conditioned on 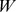, the joint measurement 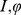 is independent with the image being a conditionally independent random field from voxel to voxel under the product distribution:
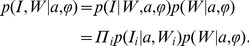
(3)The term 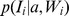 is computed using Gaussian mixture models. The probability 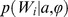 is calculated by transferring the segmentations of the atlas under the action of the diffeomorphism between the atlas and the target. For voxel 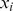
corresponding to atlas coordinate 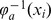 which is interior to the atlas anatomical labels so that all neighbors on the lattice are of the same label type, no interpolation is required and the prior probability is an indicator function; otherwise the probability is interpolated. To compute the joint probability of image and segmentation labeling 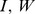 for the iterative MAP algorithm, we must solve the integral over the nuisance variables of coordinate transformations for which we use the mode approximation 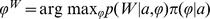 approximating
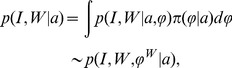
(4)with 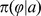 the prior on transformations conditioned on the atlas.

### 2.2.1 Local Charts

Locality is introduced into the global representations by defining the atlases to correspond to manually-labeled T1-weighted imagery with empirical mean and standard-deviation parameters obtained over the subcortical structures indexed throughout the volume. The charts are collections of the manually delineated sixteen subcortical and ventricular structures, each with means and variances; associated to each chart are the parameters 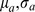 representing the structure. Locality of the atlas-charts is introduced by indexing to the target image the atlas label field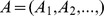, where 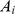 denotes the atlas-chart interpreting the target voxels.

**Figure 1:**
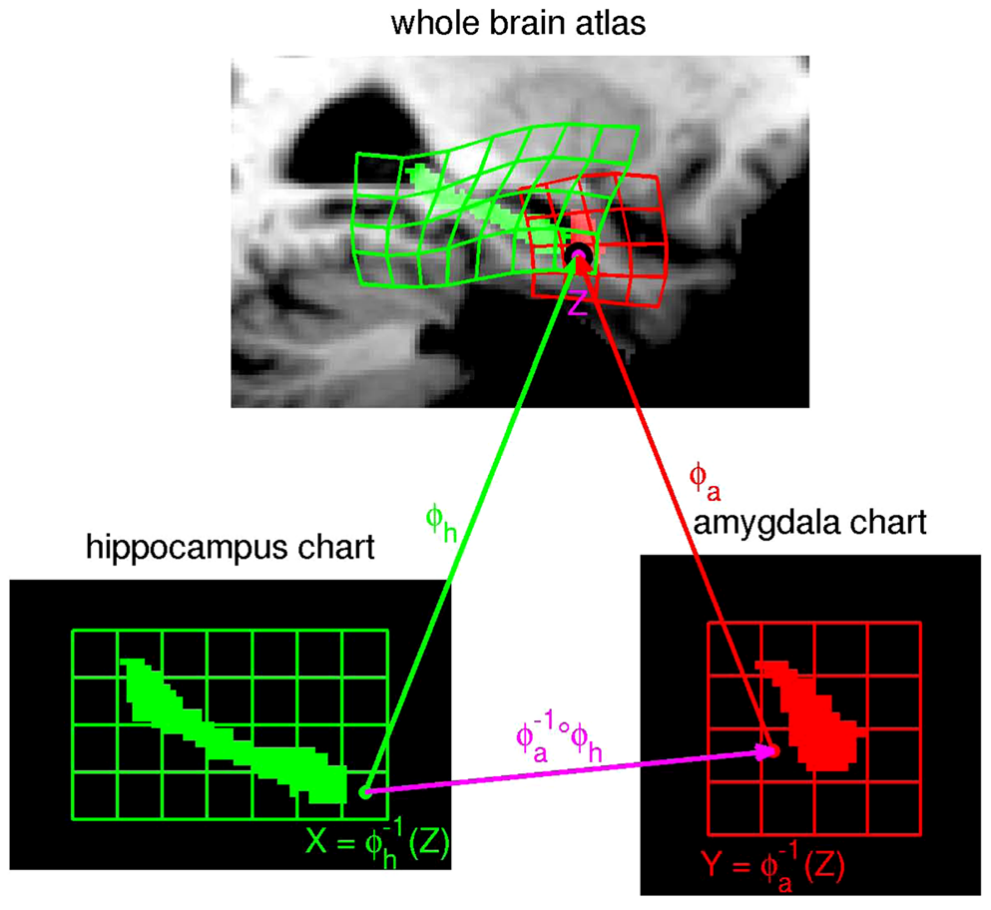
Depiction of two charts and the associated diffeomorphisms chosen to illustrate the interpretation. The charts are related via diffeomorphic coordinate transformations as depicted in the figure, in which points X, Y in the hippocampus chart and the amygdala chart are compared using the forward and inverse mappings. In our paper the charts are manually delineated structures including the amygdala, caudate, hippocampus, putamen, thalamus, lateral ventricle, the 3rd ventricle, and the 4th ventricle.

The charts are “open sets” containing each of the subcortical structure so that their unions cover the full volume and are related with each other as depicted in Figure 1 via diffeomorphic coordinate transformations. Two points
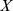
and 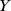
in the hippocampus chart and the amygdala chart may be compared using the forward and inverse mappings via: 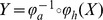, 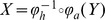. This ensures that during segmentation, multiple charts overlapping allows for weighted interpretation, since all “mediation” of errors occurs at the boundaries of the structures. At one boundary of the hippocampus, for example, are portions of the ventricles, at another the amygdala. Interpretation of those boundary voxels is supported by multiple charts which can overlap and therefore may offer alternative contributions.

The multi-atlas random orbit model of the observed imagery 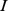 is that the mean-fields are random deformations of atlas-charts arising from perhaps different atlases, each locally indexing different parts of the brain. The image and diffeomorphism are linked through the fact that the diffeomorphism determines the segmentation.The image is conditionally Gaussian with mean determined by the deformed atlas-chart according to 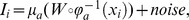 with the noise being additive Gaussian. This indexing of every point in the target image with a chart label gives the locality. The probabilistic structure we induce corresponds to splitting the image and diffeomorphism so that, given the segmentation, the image is conditionally independent of the diffeomorphism 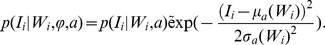 We also use Gaussian mixture models for the conditional random field as introduced in [53].

### 2.2.2 Likelihood Fusion and the EM Algorithm

We introduce the localized indicator functions associated to the atlas field labelling 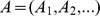 with 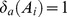 meaning that atlas 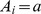 is used to interpret the image 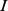; the joint density is conditionally independent between different voxels, conditioned on the atlas-chart labels given by
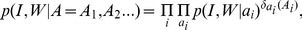
(5)where 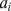 designates the atlas used to interpret the image voxels. For the case where the atlases are global, then one atlas is used to interpret the image; for all of the cases shown here the atlas-charts are locally defined subcortical structures with multiple atlas-charts interpreting each image.

#### The Algorithm

Define the Q-function as the conditional expectation of the complete-data log-likelihood according to
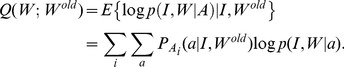
(6)


Then the sequence of iterates 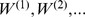, associated to the alternating maximization defined by the iteration:
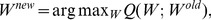
(7)is monotonic in the incomplete-data likelihood (proven in Appendix S1) with atlas selector 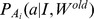. The monotonicity follows from the fact that Eq. (6) is an EM Algorithm, as proven in the Appendix S1, since
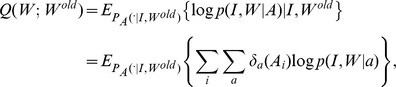
with Eq. (6) following from the expectation 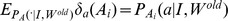. Eq. (6) is the likelihood-fusion equation. During the iteration, the sequence of conditional probabilities 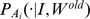 derived from the conditional mean of the indicator functions encodes the set of atlases being selected in the interpretation of any particular voxel. Computing the maximization requires calculating the integral over the nuisance of coordinate transformation for which we use the mode approximation. The general steps of the algorithm can be summarized as follows:

1. Initialize: 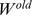, and 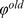
for each atlas, 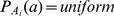

2. Compute optimized mappings
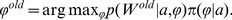
(8)
3. Compute the approximated atlas selector
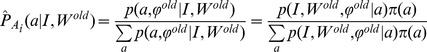
(9)
4. Generate new segmentation 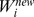, 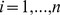 maximizing approximate Q-function
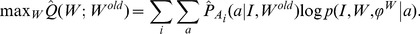
(10)
5. If either 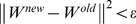 or total iterations is bigger than 100, then stop, otherwise, update segmentation,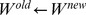, and go to 2.

#### Remark

To maximize Eq. (10), we iterate between fixing the diffeomorphism and maximizing the segmentation, then locally maximizing the diffeomorphisms for the fixed segmentation labeling using Eq. (4) to define 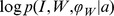. Locality is implied here since for atlas-charts, only segmentation labels in the target image in the vicinity of the atlas-chart are determined by the log-probability.

To maximize Eq. (8), we use measures of the distance between the segmentation 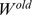
of the target structures and the diffeomorphic mapping results from the template structures to the target, analogous to the Large Deformation Diffeomorphic Metric Mapping (LDDMM) for image matching and surface matching. We have examined several approaches for computational purposes. The first computes the distance between the atlas structure and the structures in 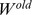 via dense LDDMM image matching [54]. Given the pair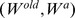, both of which are viewed as dense functions over the image domain, the vector field is generated to minimize the energy
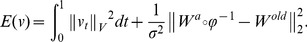
(11)


The LDDMM variational problem has direct interpretation as a MAP estimator. Associate to 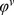 is the initial momentum or the initial vector field [55] since it satisfies the ordinary differential equation 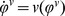. The smooth norm 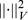 on the spatial derivatives of the velocity field is chosen via the Laplacian smoothness operator based on parameters 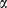 and 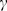, for which we use a ratio-cascading method as described in [56]; the ratio 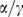 is gradually decreased to improve numerical stability and prevent sub-optimal local minima.

The second method we use to compute the distance is to create triangular meshes of the structures and compute the distance between the atlas structures and meshes of the structures in 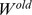 via LDDMM surface matching [57]. The third method for computing distance is to compute the overlap via set distance calculations which is extremely fast for determining 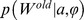; the Dice overlap is one example. For the prior probability 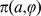, we weigh solutions via the metric distance in diffeomorphism space given by the exponential of geodesic length.

For computational purpose, we remove outlier atlases used in the computation following a robust scoring scheme analogous to that suggested in [48]. For each conditional probability representing the overlap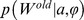, we calculate the mean 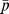 and remove atlases that are 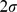 outliers:
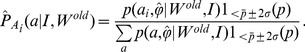
(12)


### 2.3 Subcortical Structure Segmentation

In this study, sixteen deep gray matter and ventricles structures were manually defined in the atlases images, which cover only a small part of the images. We defined a cuboid region of interest (ROI) encompassing all the structures of interest in all atlases, and modeled the segmentations within this ROI. Voxels inside the ROI not belonging to any of the sixteen manually delineated structures were automatically labeled as white matter, gray matter, or cerebrospinal fluid (CSF) based on a local brain tissue segmentation algorithm [53]. This generic labeling of tissues outside the sixteen structures of interest ensures that all voxels were labeled. Because the likelihood-fusion algorithm tries to assign a label with the highest probability to each voxel, this type of “generic” labels outside the structures of interest (in this case, the 16 manually segmented structures) is necessary to avoid over assignment.

### 2.3.1 Subject data and Comparison Metrics

In this study, we use T1-weighted images from 35 subjects from three groups, as described in Table 1. Magnetization Prepared Rapid Gradient Recalled Echo (MPRAGE) T1-WIs (TR/TE = 8.4/3.9 ms) were acquired using 3T whole-body MRI scanners (Philips Medical Systems, Best, The Netherlands), with an axial orientation and an image matrix of 256×256. Participants were scanned with two slightly different protocols: one used a field of view (FOV) of 230×230 mm and 120 slices of 1 mm thickness; and the other used an FOV of 240×240 mm and 140 slices of 1.2 mm thickness. These images were then manually segmented into sixteen structures – left and right hippocampus, amygdala, caudate, putamen, pallidum, lateral ventricle, thalamus, the 3^rd^ ventricle, and the 4^th^ ventricle.

**Table 1:** Three different groups of MR scans and their respective size, age range, resolution, imaging protocol, and pathology.

Group	Size	Age range	Resolution (mm)	Imaging protocol	Patient group
1	14	55 to 85	0.9375×0.9375×1.2	3.0T	NC elder
2	15	56 to 87	0.9375×0.9375×1.2	3.0T	AD
3	6	51 to 84	0.8984×0.8984×1.0	3.0T	Dementia (PPA)

NC indicates normal controls, AD indicates Alzheimer's disease, PPA indicates primary progressive aphasia.

To quantitatively evaluate the accuracy of our algorithm, we employed a leave-one-out cross-validation method on the datasets of Group 1 and Group 2. For Group 3, we used datasets from Group 1 and Group 2 as the atlases for segmentation. Manual segmentations were regarded as the gold standard. The segmentation accuracy was measured through the use of the Dice overlap coefficients. The Dice overlap is computed as: 
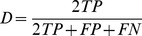
, where 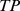, true positive, is the volume of the region that belongs to both the automated segmentation and the manual segmentation,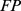, false positive, is the volume of the region that belongs to the automated segmentation but not the manual segmentation, and 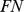, false negative, is the volume of the region that belongs to the manual segmentation but not the automated segmentation.

### Ethics Statement

All subjects provided written consent for participation in accordance under the oversight of the Johns Hopkins Medicine Institutional Review Board (JHM-IRB). Additionally, for those subjects with dementia, the consent form was co-signed by a family member. The Johns Hopkins Institutional Review Board approved this study.

### 2.3.2 Comparison with label-fusion methods

The segmentation accuracy of the proposed method was compared with that of the segmentations obtained from two label-fusion techniques: STAPLE [12] and Spatial STAPLE [58]. STAPLE and Spatial STAPLE were chosen for the comparison because they provide state-of-art segmentation accuracy and are widely used for multi-atlas label-fusion based segmentations. For a comparison, the multi-atlas LDDMM likelihood-fusion method was compared with STAPLE and Spatial STAPLE by using the codes which were available via the “MASI Label Fusion” project [58] on the NeuroImaging Informatics Tools and Resources Clearinghouse. The parameters for STAPLE and Spatial STAPLE were optimized through the consultation from Simon Warfield (STAPLE) and Bennett Landman (Spatial STAPLE). For both algorithms, we used the log-odds of the majority voting results as the explicit spatically-varing prior. The convergence factor was chosen to be 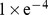. The EM algorithm for both STAPLE and Spatial STAPLE was designed to start with an initial estimate of the label probabilities, instead of the regional performance level parameters. For Group 1 and Group 2, we used the same leave-one-out testing: for each subject, the segmentation labels were transferred from the 28 atlases by the same transformation matrices derived in each single LDDMM image mapping and they were fused by STAPLE and Spatial STAPLE. For Group 3, the propagated segmentations from the 29 atlases in Groups 1 and 2 were fused.

To measure the statistical significance of differences between two groups in comparison, instead of using the student's *t*-test, we applied Fisher's method of randomization. We utilized Monte Carlo simulations to generate 10,000 uniformly distributed random permutations, which gives rise to a collection of *t*-statistics coming from each permutation. The p-value is then given by the fraction of times that the *t*-statistic values from the permutations is larger than the value obtained from the true groups.

## Results

### 3.1 Evaluation of adding neighboring generic tissue labels in the atlases

**Figure 2:**
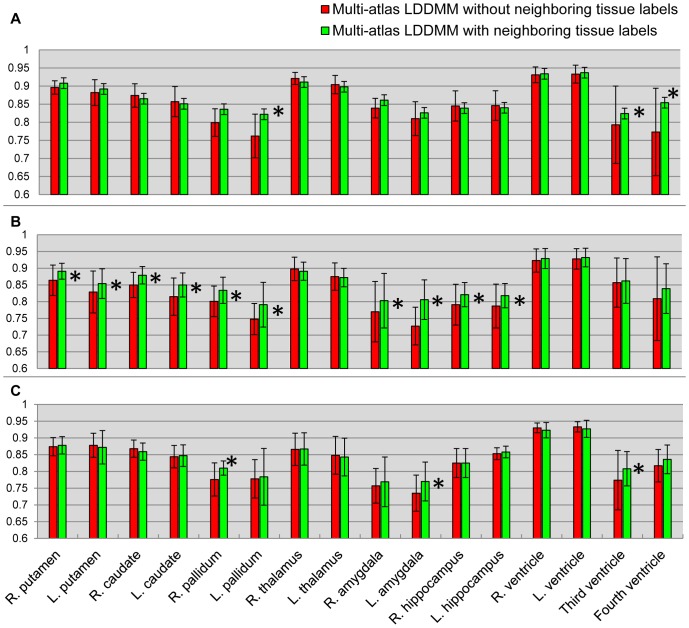
A comparison of two sets of atlas segmentations. Panels **A**–**C** show the mean Dice values and its standard deviations of the sixteen structures, for the three different groups, obtained from likelihood-fusion via multi-atlas LDDMM based on two different sets of atlas label definitions: 1) sixteen manually defined structures (red); 2) sixteen manually defined structures and three generic neighboring tissue segmentations (green).

In the first experiment, we explored the efficacy of adding neighboring tissue labels around the structures of interest (the sixteen manually delineated subcortical and ventricular structures) in the atlas. The same likelihood-fusion procedure was applied to the two sets of labels of the same atlas images: 1) the sixteen manually defined structures, and 2) the sixteen manually defined structures and the three generic neighboring tissue segmentations – gray matter, white matter, and CSF. A quantitative comparison between the two sets of automated segmentations based on the two different sets of label definitions, in terms of all the three groups, is shown in Figure 2.

As shown in Figure 2, adding neighboring tissue labels in the atlases improves the segmentation accuracy for a majority of structures, especially for subjects with dementia (Group 2 & 3). For normal subjects (Group 1), it also helps in the segmentation of certain structures such as pallidum, the 3^rd^ ventricle, and the 4^th^ ventricle. In addition to the improvements shown via average Dice values, we also observed that adding tissue definitions prevents the mislabeling between ventricles and their neighboring gray matter structure such as hippocampus and amygdala, particularly in the area close to the inferior horn.

### 3.2 Quantitative Evaluation of the benefit of Multiple Atlases

**Figure 3:**
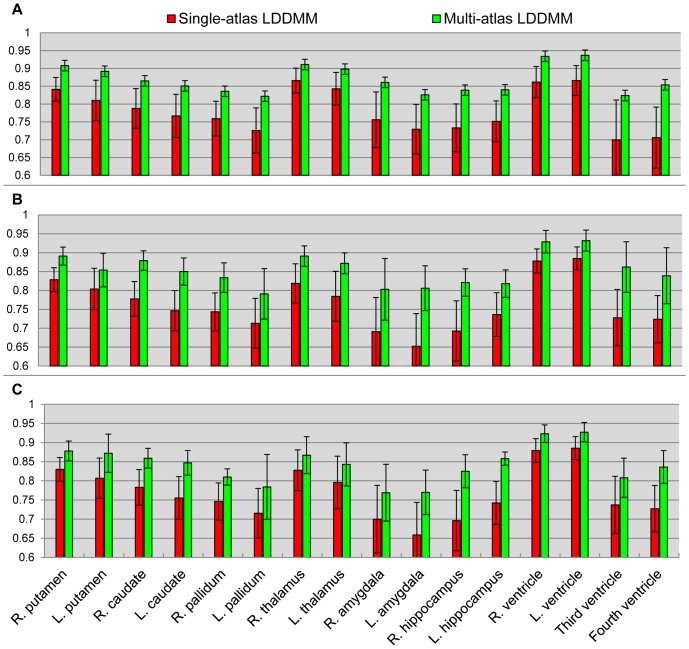
A comparison of segmentation accuracy between single-atlas LDDMM and multi-atlas LDDMM. Panels **A**, **B**, **C** respectively show the mean Dice overlaps and the standard deviations of the sixteen structures obtained from single-atlas LDDMM (red) and likelihood-fusion via multi-atlas LDDMM (green) for the three different groups.

It is clear that having multiple atlases increases the computational complexity. We wanted to be able to quantify the advantages of supporting multiple atlas anatomies in the solution. For this we performed multiple experiments. The first compares the performance of segmentation via single-atlas LDDMM using a leave-one-out technique in which a single subject was chosen as a template and all the other subjects in the group were segmented via the LDDMM image mapping procedure. For this purpose, the data in Groups 1 and 2 were combined; one of the 29 subjects was used as the atlas and the other 28 images were segmented. This process was repeated for 29 different atlases implying each subject was segmented 28 times using 28 different atlases. For subjects in Group 3, the single atlases chosen from groups 1 and 2 were used for segmentation to avoid the potential bias of the leave-one-out approach. The mean Dice values and the standard deviations for each set of Dice values for automated segmentations of various structures from single-atlas LDDMM are shown in Figure 3.

As demonstrated in Figure 3, the single-atlas LDDMM performs relatively poorly in segmenting several of the structures for the Alzheimer's disease (AD) population (Group 2), especially for the amygdala and the hippocampus. These two structures are adjacent to the inferior horn of the ventricles, which tend to have poor segmentation results due to a large topological variability and resultant LDDMM mapping inaccuracy in these areas.

**Figure 4:**
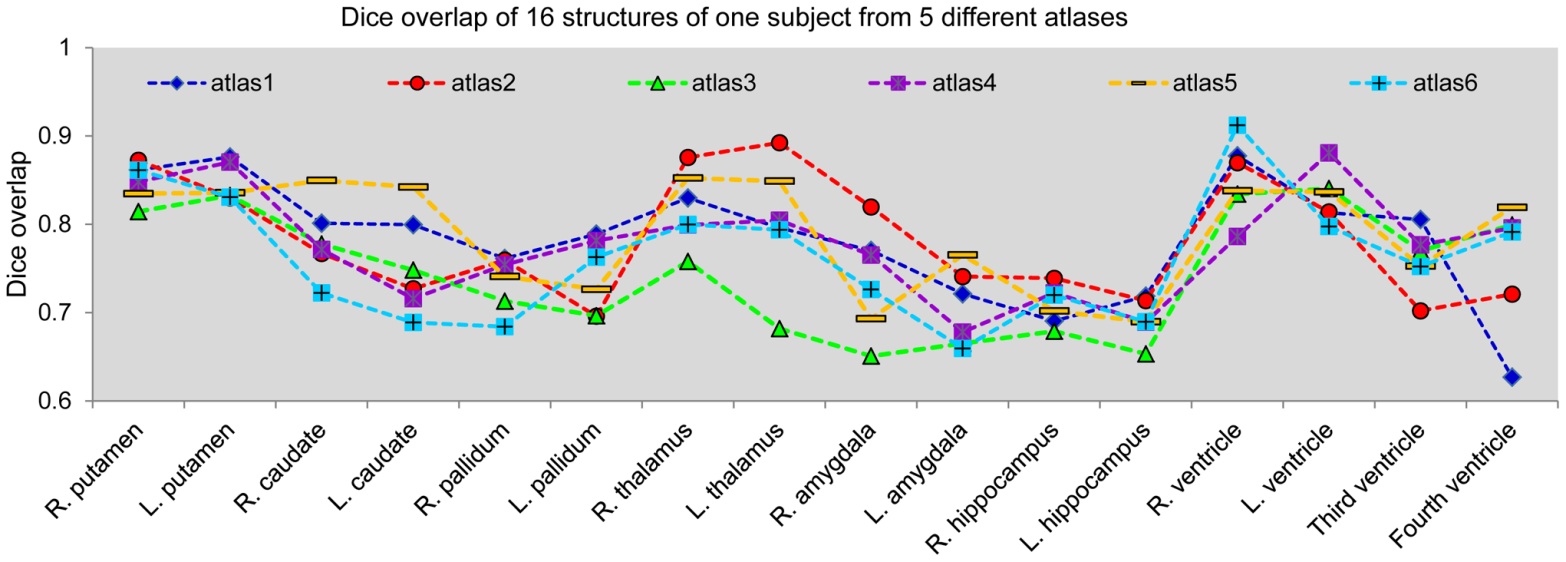
Depiction of the variability within different single atlases. Scatterplot of Dice overlaps of automated segmentations of sixteen different structures of one subject from 6 different atlases using single atlas LDDMM.

Figure 4 shows results for six representative atlases for segmentation of sixteen different structures in one subject. The figure suggests that the best atlas varies depending on the structure; there is no single atlas that outperforms all other over all sixteen structures. For example, for the segmentation of the right putamen and the thalamus in both hemispheres, atlas #2 outperformed other atlases, whereas, for the third ventricle, atlas #2 gave the lowest segmentation accuracy in terms of the Dice overlap.

**Figure 5:**
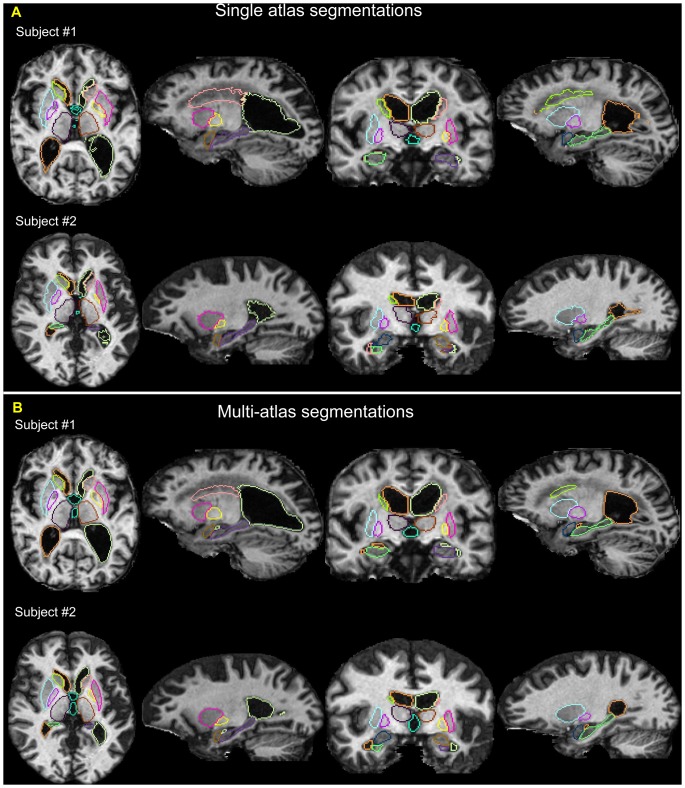
Example of subcortical segmentations from single- and multi-atlas LDDMM approaches. Panel **A** shows the automated segmentation results of two subjects using single-atlas LDDMM, while panel **B** shows the segmentation results for the same subjects using multi-atlas LDDMM approach.

To contrast to the single-atlas LDDMM case, we examined the likelihood-fusion via multi-atlas LDDMM approach using a series of leave-one-out tests combining the data from Groups 1 and 2. In the leave-one-out strategy, the remaining MRIs form the atlases. Figure 5 shows the segmentation results of two subjects for a comparison between the single-atlas and the multi-atlas approach. The Dice overlaps that resulted from multi-atlas LDDMM are also demonstrated in Figure 3 for a direct comparison with that from single-atlas LDDMM. Because of the possibility that the leave-one-out analysis using the data with a identical image protocols (Groups1 and 2 data) may not represent the real-world performance of the proposed approach, the method was applied to the Group 3 data, which were acquired with a different scanner and imaging parameters. The MRIs from Groups1 and 2 were taken as the atlases. The Dice overlap for segmentation of Group 3 using the single-atlas and multi-atlas LDDMM is also illustrated in Figure 3, demonstrating a comparable level of Dice from multi-atlas LDDMM as those obtained in Groups 1 and 2.

**Figure 6:**
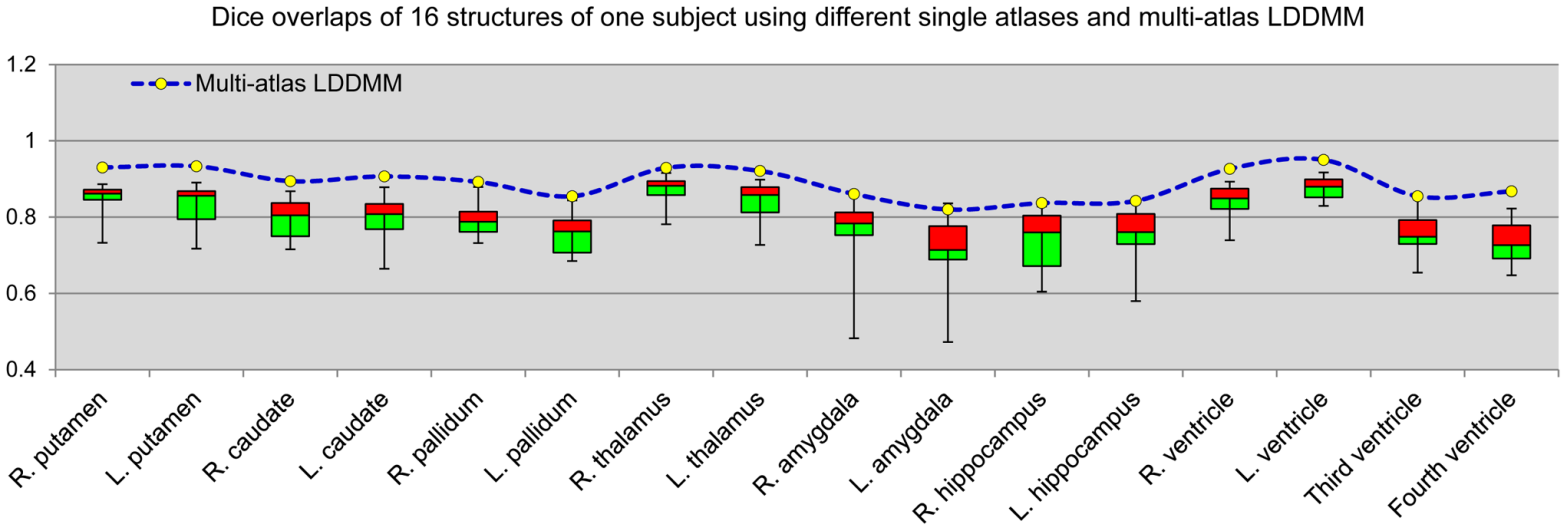
Boxplots of the Dice overlaps for sixteen different structures. The Dice overlaps were computed between the automated segmentations of sixteen different structures of one subject from 28 different atlases using single atlas LDDMM and the one from multi-atlas LDDMM (blue dotted line).

Figure 6 shows the results from one representative case, comparing Dice values of the multi-atlas approach to approaches based on selection of any of the single atlases. This figure clearly shows that likelihood-fusion via multi-atlas LDDMM form an empirical upper bound in performance even for the best combinations of the single-atlas approach for all structures. Regardless of the anatomical variability among these three populations, the multi-atlas approach consistently out-performed the single-atlas approach. For all structures in all three groups, a significant improvement in Dice values has been found with 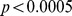 in the statistical tests.

**Figure 7:**
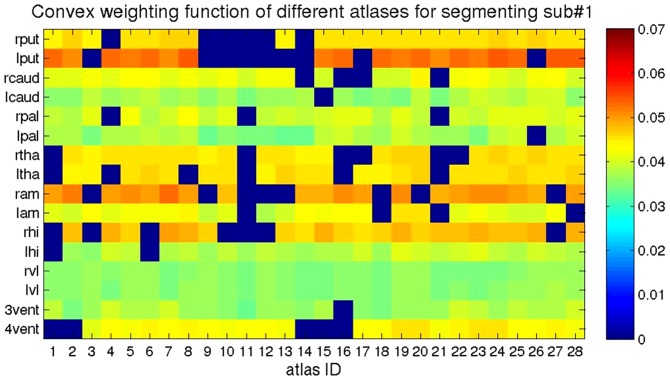
The convex weighting function normalized over each structure. For each structure, we color-coded the quantity 
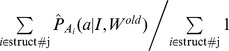
 that is depicted for each atlas (column) and each of the 16 structures (rows).

Shown in Figure 7 is an examination of the convex weighting function of Eq. (9) for segmenting one subject averaged over voxels 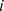 within each single structure 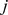, 
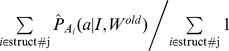
, 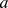 indexed as a function of atlases. As depicted in the figure different atlases contribute different weighting functions when segmenting different structures of the same subject.

**Figure 8:**
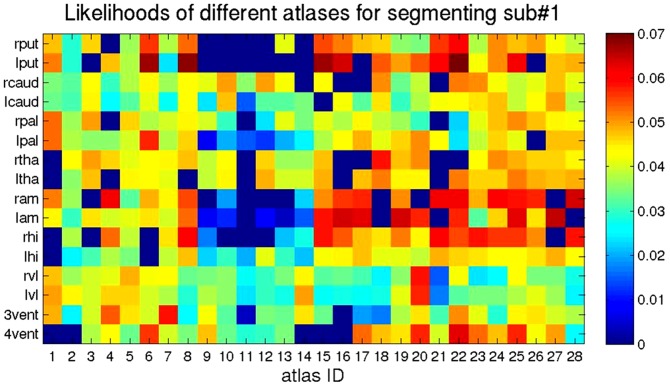
The likelihood contribution of each atlas averaged over the atlas structures. The quantity 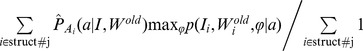 was obtained from the 28 different atlases (columns) for each structure (row).

Shown in Figure 8 is a depiction of the likelihood contribution of each atlas in the likelihood fusion equation 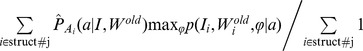 averaged over each of the 16 structures (rows) depicted for each of the twenty-eight atlases (columns).

### 3.3 Comparisons to segmentation averaging (STAPLE & Spatial STAPLE)

**Figure 9:**
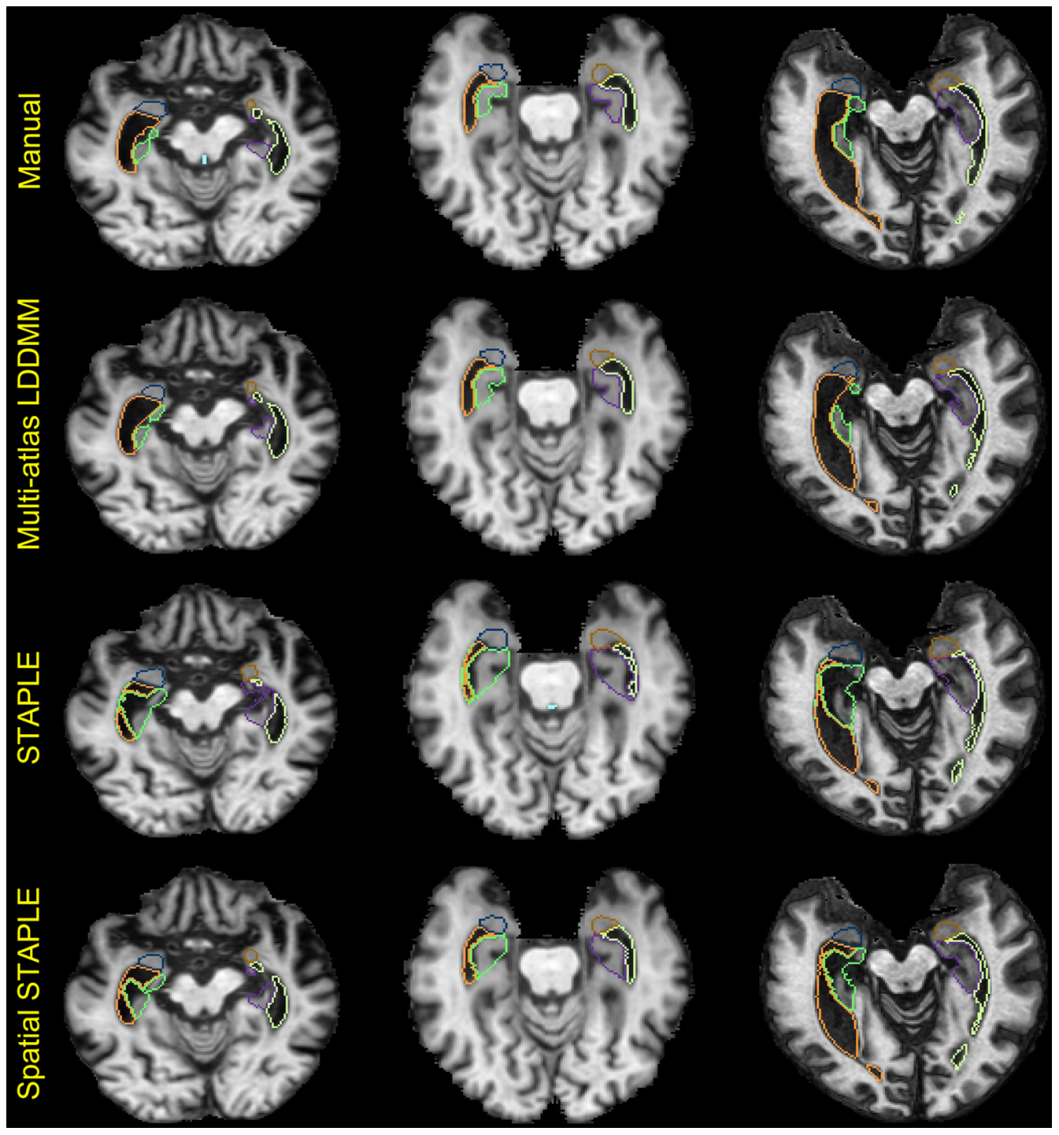
Example slices for a comparison of multi-atlas LDDMM, STAPLE, and Spatial STAPLE. Three representative 2-D slices of three structures near medial temporal regions – the amygdala, the hippocampus, and the ventricle in both hemispheres obtained respectively from manual delineation (top row), likelihood-fusion via multi-atlas LDDMM (2nd row), STAPLE (3rd row), and Spatial STAPLE (bottom row).

The generative probability model which multi-atlas LDDMM is based averages likelihoods generating a single label for each segmentation voxel. It is natural to compare to competitive methods which average segmentation labels via label fusion. For this we compared the multi-atlas LDDMM with two representative label fusion techniques, STAPLE [46] and Spatial STAPLE [58]. One might expect that while label fusion should be more robust to images for which the generative model is not accurate, likelihood fusion should provide benefits in circumstances when the generative model is valid. Tables 2–4 tabulate the mean values and standard deviations of the Dice overlaps for the three methods computed across subjects in the three groups. The performance of Spatial STAPLE and likelihood-fusion via multi-atlas LDDMM were almost identical for the control group (Table 2), providing superior performance relative to STAPLE. For the brains from patient populations, significant improvement by likelihood-fusion via multi-atlas LDDMM over Spatial STAPLE was observed for 9 structures in the AD (Table 3) and 3 structures in the primary progressive aphasia (PPA) populations (Table 4). One of the most notable improvements was found in the area around the inferior and posterior horns of the lateral ventricles, where the ventricle anatomy has a substantial amount of anatomical variability (Figure 9). The benefit must be arising from the fact even though these anatomies are disease we are able to do an adequate job of modelling the generative probability therefore the atlas selector function is effectively averaging in the proper likelihoods which fit the anatomies.

**Table 2:** The average Dice overlaps between manual volume and the automated volume measured over the fourteen datasets of the first group for each structure for comparisons of STAPLE, Spatial STAPLE, and likelihood-fusion via multi-atlas LDDMM.

	STAPLE	Spatial STAPLE	multi-atlas LDDMM
R. putamen	0.878 (0.0250)	**0.908 (0.0154)**	**0.908 (0.0148)**
L. putamen	0.857 (0.0362)	**0.891 (0.0350)**	**0.892 (0.0329)**
R. caudate	0.836 (0.0409)	**0.867 (0.0355)**	**0.865 (0.0446)**
L. caudate	0.812 (0.0506)	**0.852 (0.0365)**	**0.851 (0.0465)**
R. pallidum	0.776 (0.0357)	**0.840 (0.0291)**	**0.836 (0.0378)**
L. pallidum	0.730 (0.0543)	**0.817 (0.0501)**	**0.822 (0.0463)**
R. thalamus	0.907 (0.0224)	0.911 (0.0183)	0.911 (0.0185)
L. thalamus	0.883 (0.0370)	0.906 (0.0246)	0.898 (0.0199)
R. amygdala	0.786 (0.0401)	**0.862 (0.0245)**	**0.861 (0.0188)**
L. amygdala	0.767 (0.0527)	**0.822 (0.0393)**	**0.826 (0.0388)**
R. hippocampus	0.769 (0.0461)	**0.838 (0.0337)**	**0.839 (0.0297)**
L. hippocampus	0.795 (0.0499)	**0.846 (0.0278)**	**0.840 (0.0227)**
R. ventricle	0.874 (0.0465)	**0.917 (0.0267)**	**0.934 (0.0247)**
L. ventricle	0.880 (0.0503)	**0.918 (0.0336)**	**0.937 (0.0259)**
Third ventricle	0.548 (0.1712)	0.776 (0.1214)	**0.824 (0.0817)**
Fourth ventricle	0.671 (0.1287)	0.799 (0.0931)	**0.854 (0.0776)**

Bold typesetting indicates that the Dice overlap ratio obtained from the corresponding is statistically significant higher than that of other methods (*p<0.05*).

**Table 3:** Mean and standard deviations of Dice overlaps computed across the fifteen subjects in the second group for each structure for comparisons of STAPLE, Spatial STAPLE, and likelihood-fusion via multi-atlas LDDMM.

	STAPLE	Spatial STAPLE	multi-atlas LDDMM
R. putamen	0.838 (0.0504)	0.878 (0.0351)	**0.891 (0.0238)**
L. putamen	0.813 (0.0661)	**0.848 (0.0525)**	**0.854 (0.0444)**
R. caudate	0.776 (0.0558)	0.856 (0.0346)	**0.879 (0.0260)**
L. caudate	0.746 (0.0696)	**0.828 (0.0454)**	**0.850 (0.0359)**
R. pallidum	0.784 (0.0547)	**0.831 (0.0389)**	**0.834 (0.0392)**
L. pallidum	0.746 (0.0475)	**0.792 (0.0522)**	**0.791 (0.0668)**
R. thalamus	0.881 (0.0355)	0.900 (0.0313)	0.891 (0.0270)
L. thalamus	0.855 (0.0541)	0.876 (0.0381)	0.872 (0.0275)
R. amygdala	0.703 (0.0974)	**0.796 (0.0948)**	**0.803 (0.0816)**
L. amygdala	0.647 (0.0708)	0.785 (0.0647)	**0.806 (0.0592)**
R. hippocampus	0.670 (0.0988)	0.783 (0.0759)	**0.821 (0.0362)**
L. hippocampus	0.703 (0.0859)	0.799 (0.0556)	**0.818 (0.0363)**
R. ventricle	0.904 (0.0481)	0.917 (0.0326)	**0.929 (0.0299)**
L. ventricle	0.908 (0.0466)	0.921 (0.0333)	**0.932 (0.0278)**
Third ventricle	0.678 (0.1244)	0.830 (0.0426)	**0.862 (0.0668)**
Fourth ventricle	0.706 (0.1245)	0.793 (0.0862)	**0.839 (0.0741)**

Bold typesetting indicates that the Dice overlap ratio obtained from the corresponding is statistically significant higher than that of other methods (*p<0.05*).

**Table 4:** Mean and standard deviations of Dice overlaps obtained respectively from STAPLE, Spatial STAPLE, and likelihood-fusion via multi-atlas LDDMM in segmenting the 16 structures of the six subjects in the third group.

	STAPLE	Spatial STAPLE	multi-atlas LDDMM
R. putamen	0.866 (0.0297)	0.884 (0.0166)	0.878 (0.0260)
L. putamen	0.856 (0.0392)	0.878 (0.0413)	0.872 (0.0499)
R. caudate	0.861 (0.0327)	0.854 (0.0253)	0.859 (0.0258)
L. caudate	0.832 (0.0432)	0.836 (0.0218)	0.847 (0.0322)
R. pallidum	0.788 (0.0393)	0.817 (0.0261)	0.810 (0.0212)
L. pallidum	0.763 (0.0520)	0.777 (0.0686)	0.784 (0.0847)
R. thalamus	0.871 (0.0298)	0.854 (0.0494)	0.867 (0.0482)
L. thalamus	0.849 (0.0415)	0.828 (0.0546)	0.843 (0.0563)
R. amygdala	0.707 (0.0568)	0.769 (0.0668)	0.769 (0.0741)
L. amygdala	0.662 (0.0752)	**0.745 (0.0561)**	**0.770 (0.0580)**
R. hippocampus	0.777 (0.0987)	0.796 (0.0385)	**0.825 (0.0432)**
L. hippocampus	0.805 (0.0251)	0.839 (0.0236)	**0.858 (0.0174)**
R. ventricle	0.927 (0.0144)	0.924 (0.0192)	0.923 (0.0229)
L. ventricle	0.927 (0.0246)	0.926 (0.0201)	0.927 (0.0255)
Third ventricle	0.749 (0.1034)	0.803 (0.0327)	0.808 (0.0511)
Fourth ventricle	0.738 (0.0487)	0.811 (0.0292)	**0.836 (0.0428)**

Bold typesetting indicates that the Dice overlap ratio obtained from the corresponding is statistically significant higher than that of other methods (*p<0.05*).

## Discussion

As accurate segmentation is at the center of many neuropsychiatric studies, there have been many methods developed for brain segmentation, which are typically based on local approaches mostly involving multi-compartment appearance and Gaussian mixture modeling, coupled to MAP or maximum-likelihood [53], [59]–[69]. To introduce constraints between voxels, Markov random fields and level sets are two examples of locally-defined prior distributions enforcing interactions at the voxel level [1], [70]–[78]. Similar appearance modeling is used in the deformable template approaches as the matching cost functions; the higher lever relationships are inherited from the templates. The MAP segmentation we used is a direct generalization of the MAP approach originally articulated by [1] in which global constraints are introduced via Markov random field conditional probability structure at the segmentation layer. The approach here is based on the diffeomorphic orbit model to induce neighborhood dependence at the random field level.

The conditionally Gaussian random field model used throughout is the generative random orbit model used for “template” estimation [39], [79], [80]. Whereas, for template estimation, the template and the diffeomorphism of the hyper-template are the unknown, and the population contributes through the conditional likelihood functions associated to each of the multiple anatomical coordinate systems in the atlas set. In this paper, the segmentation field plays the role of the unknown, and the population is represented via the charts in the atlases.

In these global deformable template methods, templates which are far in the deformation space are less accurate for representing anatomical features and parameters being estimated. In the context of segmentation, multiple atlas based methods which embed the global solution with more locally accurate properties via label combination have been used extensively [37], [46]. The multi-label interpretation approach, as described in [46], enters into our method only indirectly, as we interpret each voxel position in the anatomical target subject as arising from any of the given atlases. Therefore, this must be interpreted by the Bayesian conditional probability of each atlas chart contribution conditioned on the image. The method described here fuses the atlases via convex combination of the atlas-specific likelihoods, with the weights in the convex combination given by the conditional-mean formula, and never explicitly generates the atlas-specific segmentations of the target MRI. The purpose of the conditional-mean framework of the EM algorithm is to remove the explicit dependence of the estimation of the target segmentation on the high dimensional nature of the nuisance variables. It serves the same purpose as in [39] and [80] – the nuisance fields do not grow with the number of atlases, which could have the disadvantage that it would make segmentation of the target MRI inconsistent.

Another aspect of the diffeomorphic framework is that since we model human anatomy via the diffeomorphism metric as a complete metric space [35], [55], [81], [82], our weighting in the MAP solution is specified via the metric distance between atlases and the subject. Similar to the method proposed in [48], this allows us to introduce a robust decision procedure, which decreases the computational complexity by removing atlases that are large in metric distance.

The proposed method has been tested based on three datasets with different pathologies – normal aging, subjects with Alzheimer's disease, and subjects with dementia. Likelihood-fusion via multi-atlas LDDMM improves the segmentation accuracy obtained from single-atlas LDDMM. Favorable comparison to label-fusion methods is also evident as shown in Tables 2–4.

Compared with other recently published segmentation methods and the reported Dice overlaps, our method demonstrates comparable or favorable levels of segmentation accuracy, with mean Dice overlap results in the range of 0.8 to 0.93. A direct comparison of segmentation accuracy among different programs is difficult as many programs contain internal structural definition with the resultant differences in performance which can simply reflect the way that structures are defined. Given the structure sizes and the intensity ranges, it is generally considered more difficult to automatically segment the hippocampus and amygdala than other deep gray matter structures. Previous publications such as [83] have reported Dice values such as 0.73 and 0.76 for the hippocampus, and [84] has reported Dice overlaps on the order of 0.75 for the amygdala using either FreeSurfer [1] and FSL [69]. In the most recent work specifically on the segmentation of hippocampus [85], reported Dice of 0.88 for the best performing subjects while 0.78 for the worst subjects. [86] reported hippocampus segmentations with mean Dice 0.83. Our results compare favorably, although it is difficult to directly compare Dice values from different studies given the difference that may be caused by the dataset used, the image acquisition protocol, or the quality and the protocol of manual segmentations. One future direction should be evaluating the proposed method on some more widely studies datasets so as to be comparable with other existing segmentation methods [86], [87]. We have chosen to focus our study on populations with severe atrophy and the reported Dice values should represent more realistic performance than those based only on young healthy subjects such as those reported in [85]. As shown in Figure 5, in addition to the accuracy reports, the likelihood-fusion approach in the diffeomorphic setting exhibits smooth boundaries for the segmentations, which is not typical in the usual intensity-based segmentation approaches.

The current work has focused on subcortical and ventricular regions. Our initial investigation into whole brain segmentation setting via likelihood-fusion has been validated in a limited setting in [88]. We might expect that the very simple model of conditionally Gaussian (essentially single compartment modeling of the intensity) can be significantly improved via the incorporation of multi-compartment mixture modeling such as in [53]. In addition, the results presented in this paper only make use of T1-weighted images. Incorporating multi-modality data (T2, diffusion) information into our approach should increase the segmentation accuracy. A clear potential limitation of this method is that it requires manual labeling of multiple atlases, which is more labor-intensive compared to the single-atlas approach, and increases the computational complexity by 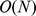, where 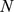
denotes the number of atlases.

## Supporting Information

Appendix S1 **Proof of the monotonicity in the incomplete-data likelihood with the atlas selector.**
(DOCX)
