# Supplementary material for: Bayesian Parameter Estimation and Segmentation in the Multi-Atlas Random Orbit Model
Source: PLoS One. 2013 Jun 18;8(6):e65591. doi: 10.1371/journal.pone.0065591 (PMC3688886; doi:10.1371/journal.pone.0065591)
Supplement: Appendix S1 — Proof of the monotonicity in the incomplete-data likelihood with the atlas selector. (DOCX) [file pone.0065591.s001.docx]

**Appendix**

**Statement:** Given the maximum a posteriori (MAP) problem

$\max_{W=W_{1},...,W_{N}}\sum_{a} p(I,W|a)\pi(a)$, (1)

then the mapping $W^{old}\to W^{new}$ with atlas selector $P_{A}(a|I,W^{old})$, associated to iteration

$W^{new}=\mathrm{argmax}_{W}\sum_{a} P_{A}(a|I,W^{old})\log p(I,W|a)$, (2)

has fixed points maximizing Eq. (1) and is monotonically increasing in likelihood with

$\sum_{a} p(I,W^{new}|a)\pi(a)\geq\sum_{a} p(I,W^{old}|a)\pi(a)$. (3)

**Proof:** Maximizing (2) with respect to parameters $\theta=W$ and evaluating at fixed points, $W^{old}=W^{new}=W^{*}$gives,

$\begin{matrix} 0\text{ }=\text{ }\partial_{W}\sum_{a} P_{A}(a|I,W^{old})\log p(I,W|a)|_{W^{*}}=\sum_{a} \frac{p(I,W^{old}|a)}{p(I,W^{old})}\pi(a)\frac{\partial_{W}p(I,W|a)}{p(I,W|a)}|_{W^{*}} \\ \text{ = }\sum_{a} \frac{\partial_{W}p(I,W^{*}|a)}{p(I,W^{*})}\pi(a). \end{matrix}$ . (4) The monotonicity is proven showing it is an EM algorithm [41]. The image augmented with uknown atlas labellings is the complete-data $(I,A)$ with incomplete-data the MRI observable$I$, and the many-to-one map $h:(I,A)\to I$discards the labels. The atlases generating the image voxels are not observable; the conditional expectations of the indicator functions determine how to fuse the likelihood interpretation associated to each atlas. Introducing the indicator function $\delta_{A}\left(a\right)=1$ means atlas $A=a$ generates the image, the density conditioned on the complete-data atlas labels is given by

$p(I,W|A)=\prod_{a} p(I,W|a)^{\delta_{A}(a)}$. (5)

Computing the logarithm and taking the conditional mean expectation of the complete-data log-likelihood gives the weighting of the log-likelihood

$\begin{matrix} E\left\{\log p(I,W)|I,W^{old}\right\}=\sum_{a} E\left\{1_{A}(a)|I,W^{old}\right\}\log p(I,W|a) \\ \text{ =}\sum_{a} P_{A}(A=a|I,W^{old})\log p(I,W|a) \end{matrix}$, (6)

where we have used $E\left\{1_{A}(a)|I,W^{old}\right\}=P_{A}(a|I,W^{old})$ proving the iteration. The monotonicity is proved using the properties of EM Algorithms. Define the complete-data $X=(I,A)$ and its likelihood$f(X;\theta)$given by Eq. (5); the incomplete-data is denoted as $Y=I$ with likelihood $g(Y;\theta)$ of Eq. (1). The many-to-one map discards the labels so that the likelihoods transform according to$g(Y;\theta)=\int_{\left\{X:h(X)=Y\right\}} f(X;\theta)dX$. Denoting the conditional density as $k(X|Y;\theta)=\frac{f\left(X;\theta\right)}{g(Y;\theta)}$, then 1-step of the mapping gives:

$E_{Y,\theta^{old}}\log\frac{f\left(X;\theta^{old}\right)}{k(X|Y;\theta^{old})}\leq E_{Y,\theta^{old}}\log\frac{f\left(X;\theta^{new}\right)}{k(X|Y;\theta^{old})}\text{ }\leq\max_{p}\int_{\left\{X:h(X)=Y\right\}} p(X)\log\frac{f\left(X;\theta^{new}\right)}{p(X)}dx$. (7)

The cross-entropy maximizer is given by the tilted distribution $\hat{p}(X)=\frac{f(X;\theta^{new})}{g(Y;\theta^{new})}$ introduced by [89]. Since this is the conditional density, the left and right hand sides of Eq. (7) are none other than the incomplete data log-likelihoods giving $\log g(Y;\theta^{old})\leq\log g(Y;\theta^{new}).$

QED
